# Engineering Photosynthetic Bioprocesses for Sustainable Chemical Production: A Review

**DOI:** 10.3389/fbioe.2020.610723

**Published:** 2021-01-08

**Authors:** Sheida Stephens, Radhakrishnan Mahadevan, D. Grant Allen

**Affiliations:** ^1^Department of Chemical Engineering and Applied Chemistry, University of Toronto, Toronto, ON, Canada; ^2^Institute of Biomedical Engineering, University of Toronto, Toronto, ON, Canada

**Keywords:** purple bacteria, cyanobacteria, metabolic engineering, biofilm, photosynthesis, bioprocess, waveguide, solar energy

## Abstract

Microbial production of chemicals using renewable feedstocks such as glucose has emerged as a green alternative to conventional chemical production processes that rely primarily on petroleum-based feedstocks. The carbon footprint of such processes can further be reduced by using engineered cells that harness solar energy to consume feedstocks traditionally considered to be wastes as their carbon sources. Photosynthetic bacteria utilize sophisticated photosystems to capture the energy from photons to generate reduction potential with such rapidity and abundance that cells often cannot use it fast enough and much of it is lost as heat and light. Engineering photosynthetic organisms could enable us to take advantage of this energy surplus by redirecting it toward the synthesis of commercially important products such as biofuels, bioplastics, commodity chemicals, and terpenoids. In this work, we review photosynthetic pathways in aerobic and anaerobic bacteria to better understand how these organisms have naturally evolved to harness solar energy. We also discuss more recent attempts at engineering both the photosystems and downstream reactions that transfer reducing power to improve target chemical production. Further, we discuss different methods for the optimization of photosynthetic bioprocess including the immobilization of cells and the optimization of light delivery. We anticipate this review will serve as an important resource for future efforts to engineer and harness photosynthetic bacteria for chemical production.

## Introduction

Societal dependence on fossil fuels is extensive and commonplace. This can be inferred from their widespread use in diverse applications including power, transportation, heating, and in the manufacture of plastics and beauty products. In an analysis using 2013 data, it was estimated that over 800 megatons of the chemical products we use annually are produced using petrochemical feedstocks (Levi and Cullen, 2018). Considering fossil fuels as a resource are finite and its extraction and use is a known contributor to the accumulation of greenhouse gases, finding viable alternative energy sources and chemical feedstocks is a challenge facing this generation.

Solar energy, as the most abundant energy source available on Earth (Blankenship et al., 2011), usually elicits images of large expanses of buzzing, black panels of photovoltaic cells which convert the energy from solar rays into electricity. However, the world’s oldest photovoltaic cells evolved within living organisms— the photosystems of photosynthetic bacteria capture photons to provide themselves the energy required for survival. Harnessing their ability to harvest solar energy and using it to produce the chemicals humans use daily could turn these bacteria into solar-powered microbial cell factories. If chemicals could be produced through this method at high enough yields and rates to be economically viable, a new era of consumerism could be ushered in— one that is both carbon-neutral and completely renewable.

Finding new pathways in photosynthetic organisms to convert substrates to valuable products is an area that has garnered a great deal of interest in recent years (Scholes et al., 2011; Bensaid et al., 2012; Carroll et al., 2018; Knoot et al., 2018; Hitchcock et al., 2020; Tiang et al., 2020). While processes using alternative substrates are also being developed in non-photosynthetic microbes, the most widely used carbon feedstock industrially is glucose from molasses or starch (Wendisch et al., 2016). Feedstock costs constitute most of the operating costs of a biological process and, on a large scale, this can be prohibitive unless the product value is high (Connelly et al., 2015). Alternatively, anthropogenic CO_2_ or wastewater streams could be used as the feedstock to synthesize chemicals using photosynthetic organisms. Considering large companies pay for the wastewater treatment of their processes and for the offset of their carbon emissions, using them as inputs to bioprocesses could have the potential to be both economically and ecologically lucrative in that they would both treat waste and output a product.

Typically, “photosynthesis” refers to oxygenic photosynthesis: the process performed by plants, algae, and cyanobacteria that generates oxygen through water splitting. This process is credited to having caused the oxygenation of Earth that made multicellular life possible over 500 million years ago (Madigan et al., 2010a) and continues to do so to this day. However, this process is theorized to have evolved 3.5 billion years ago from the lesser-known anoxygenic photosynthesis, a form that does not produce oxygen (Blankenship et al., 2007; Madigan et al., 2010a; Allen and Williams, 2011). Cyanobacteria are the only bacteria that perform oxygenic photosynthesis and they are an appealing chassis for biotechnological applications due to their genetic tractability particularly when compared to organisms like plants and algae (Jensen and Leister, 2014; Knoot et al., 2018; Hitchcock et al., 2020). Purple non-sulfur bacteria (PNSB) perform anoxygenic photosynthesis and are attractive for engineering because of their anaerobicity, metabolic versatility, and ability to naturally produce hydrogen (Tiang et al., 2020). Also, because they have been used as a model for the study of photosynthesis, their photosystems are generally well-understood (Yang et al., 2017; Fixen et al., 2019).

This review will look at both oxygenic and anoxygenic photosynthesis focusing on cyanobacteria and PNSB and consider how the different photosynthetic systems are being used and engineered both on a genetic level and a bioprocess level to overproduce native chemicals or to divert electrons toward heterologous products. When developing a bioprocess, an engineer must take a holistic approach: from organism selection based on biological strengths, to considerations of internal cellular processes, and finally to the design of the overall process. The electron transport chains of oxygenic and anoxygenic photosynthesis are similar but have several distinctions that would serve different applications. The complexes that drive photosynthesis can be engineered for increased electron flux to downstream cellular processes such as for the production of chemicals through the introduction of heterologous pathways. Additionally, external factors such as photobioreactor design can affect production as cells react to their environment and, as such, is a significant consideration. This review will examine each of these areas with the overarching goal of bioprocess design for solar-powered cell factories.

## A Comparison of Oxygenic and Anoxygenic Photosynthesis

**FIGURE 1:**
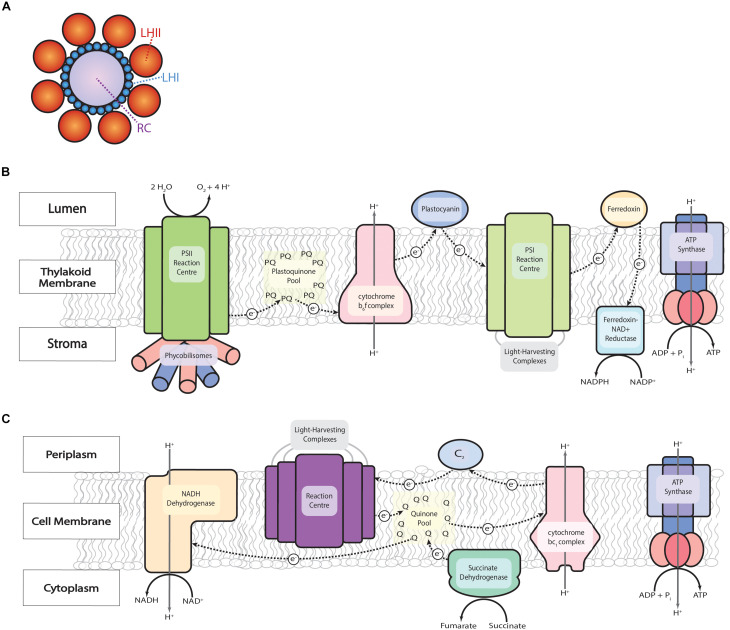
The ETC of oxygenic and anoxygenic photosynthesis. **(A)** Arrangement of light harvesting complexes (LH) around the reaction center (RC) of photosynthetic bacteria with two types of light harvesting complexes. Based on an atomic force micrograph of *Phaeospirillum molischianum* (Fassioli et al., 2009). **(B)** In oxygenic photosynthesis, as in cyanobacteria, there is a two-stage process of electron excitation. Solar energy is absorbed by the light-harvesting antenna complexes that surround the reaction center of Photosystem II (PSII) and transferred by resonance energy transfer to its reaction center where water is split, oxygen is released, hydrogen protons create a proton gradient across the membrane, and electrons begin to travel down the ETC first to the plastoquinone (PQ) pool then to the cytochrome b_6_f complex. The cytochrome b_6_f complex pumps more protons across the membrane and transfers electrons to Photosystem I (PSI) via plastocyanine and then transferred to ferredoxin- NADP^+^ reductase via ferredoxin to reduce NADP^+^ to NADPH. **(C)** Anoxygenic photosynthesis, as in PNSB, is simpler than that of oxygenic photosynthesis as there is only one reaction center for the excitation of electrons (e^–^) and the flow of these electrons is cyclic. The light-harvesting complexes accept photons and transfer energy to the reaction center where electron excitation occurs and begins to travel along the ETC to quinone (Q), cytochrome bc_1_, and cytochrome c_2_ (c_2_) generating a proton motive force whereby ATP can be synthesized via ATP synthase. PNSB benefits from a large buildup of an electrochemical energy that creates a reverse electron flow from the quinone pool to NADH dehydrogenase to produce NADH from NAD^+^.

From a high-level perspective, photosystems can be likened to photovoltaic cells: solar energy packets known as quanta (Madigan et al., 2010b) are converted into electrical energy within photosystems, transferred along the electron transport chain (ETC), and ultimately stored in chemical bonds in energy carriers like ATP and NADH or NADPH for later use. The photosystem consists of light-harvesting complexes arranged around a reaction center similar in appearance to petals around a sunflower and in function to an antenna (Figure 1A). The reaction center is where solar energy is used to excite an electron and initiate the action of the ETC, but the majority of energy needed to do this is transferred from the antenna, which increases the cross-section for light absorption by approximately 100 times (Scholes et al., 2011).

Each reaction center is surrounded by a variety of nearly 200 choromophores (colored pigments like carotenoids, phycobilins, and chlorophyll or bacteriochlorophyll) bound in light harvesting complexes (Scholes et al., 2011). Different organisms contain different combinations of chromophores and, therefore, different ranges of absorbable light wavelengths, depending on their evolutionary history (Arp et al., 2020). In addition to their functionality in energy absorption for photosynthesis, these pigments are marketable products for dyes and as health products. For instance, carotenoids are brightly colored red and orange pigments that give foods such as tomatoes and carrots their characteristic color. Photosynthetic cells use them for the absorption of short wavelength, high energy light, protection from light damage, and as antioxidants (Hashimoto et al., 2016). Carotenoids also function as antioxidants when consumed by humans, making them marketable health supplements and beauty products, of which the most well-known is β-carotene (Stahl and Sies, 2003).

Oxygenic photosynthesis (Figure 1B) has a two-stage process of electron excitation found in plants, algae, and cyanobacteria. Solar energy is absorbed by the antenna-like light-harvesting complexes that surround the reaction center of Photosystem II (PSII) and transferred by resonance energy transfer to its reaction center where this energy is used to break the O-H bond in water, releasing molecules of dioxygen (O_2_), protons (H^+^), and electrons (e^–^). The electrons begin to travel down the ETC and the protons create an electrochemical gradient across the thylakoid membrane. First, the electrons are transferred to a pool of plastoquinone (PQ), reducing it to plastoquinol (PQH_2_). PQH_2_ can then diffuse through the membrane to transfer electrons to the cytochrome b_6_f complex. Ultimately, one electron ends up being transferred to photosystem I (PSI) by diffusion of either cytochrome c_6_ or plastocyanine depending on the organism expression (Lea-Smith et al., 2016). Once in PSI, the electron is re-excited by the absorption of solar energy. The electron is then transferred by diffusion of ferredoxin to reduce NADP^+^ via the enzyme ferredoxin-NADP^+^ reductase (Lea-Smith et al., 2016).

Anoxygenic photosynthesis (Figure 1C) such as that performed by PNSB is arguably a simpler process with only one stage of electron excitation and cyclic electron flow to generate the proton motive force necessary for ATP production. Just as in the oxygenic process, solar energy is absorbed by the light-harvesting complexes and transferred to the reaction center to excite an electron, but this electron is then transferred to the quinone pool. From there, electron pairs are transferred to the cytochrome bc_1_ complex to pump protons across the membrane, then on to cytochrome c_2_, and back to the reaction center in a cyclic process to generate a proton motive force that drives ATP production. Another differentiation from oxygenic photosynthesis is that there can be reverse electron flow in anoxygenic photosynthesis when there is sufficient build-up in the quinone pool: electrons travel against the thermodynamic gradient, driven by the energy of the proton motive force toward NADH dehydrogenase to generate NADH from NAD^+^ (Madigan et al., 2010b; Adessi and De Philippis, 2013).

Anoxygenic photosynthesis may only have one stage of light harvesting but it requires less ubiquitous, more reduced molecules than water as an electron donor, the donor for oxygenic photosynthesis. However, PNSB are widely considered among the most metabolically diverse groups of bacteria (Larimer et al., 2004; Pechter et al., 2016; Imhoff, 2017). Using light as their energy source and organic carbons as both the electron donor and carbon source, photoheterotrophy is the preferred mode of growth but PNSB can switch between any of the metabolic modes that support life: photoautotrophy, photoheterotrophy, chemoautotrophy, and chemoheterotrophy (Imhoff, 2017). This metabolic versatility is one aspect that makes it attractive for engineering not only because of the breadth of opportunities for products but also because of the possibilities for feedstocks. PNSB have been studied extensively for wastewater cleanup (Adessi et al., 2017) including in olive mill wastewater (Eroglu et al., 2010; Pintucci et al., 2013), beet molasses (Keskin and Hallenbeck, 2012), dairy wastewater (Seifert et al., 2010b), soy sauce wastewater (Anam et al., 2012), brewery wastewater (Seifert et al., 2010a), a swine wastewater ditch (Okubo et al., 2006), and crude glycerol (Sabourin-Provost and Hallenbeck, 2009; Ghosh et al., 2012a, b). It has also been shown to be effective for the bioremediation of aromatic compounds (Harwood and Gibson, 1986, 1988; Sasikala and Ramana, 1998; Zengler et al., 1999; Gibson and Harwood, 2006) including lignin monomer degradation (Harwood and Gibson, 1988; Salmon et al., 2013).

The origin of photosynthesis and how it has evolved is a subject that has been studied for decades and has yet to reach definitive conclusions (Hohmann-Marriott and Blankenship, 2011; Cardona, 2019). Based on phylogenetic analysis, the current consensus is that anoxygenic photosynthesis preceded oxygenic photosynthesis (Xiong and Bauer, 2002) and that oxygenic photosynthesis evolved from anoxygenic photosynthesis (Dismukes et al., 2001). Whichever way photosynthesis evolved, the organisms operating on it often evolved in the environment together (Cogdell et al., 2006). In lakes, cyanobacteria and algae are often aggregated near the surface where the water is aerobic and where their chlorophyll absorb a large portion of the blue and red light. Whereas purple bacteria can be found beneath them in the microaerobic to anaerobic depths and where the solar spectrum has been filtered by the upper level microbes leaving the longer wavelength green and red light to be absorbed by carotenoids and bacteriochlorophyll, respectively (Cogdell et al., 2006). The specific pigments and carotenoids that make up the antenna complexes of photosynthetic organisms evolved based on light availability in their environmental niche. Using a network model, the ideal absorption wavelengths of bacterial photosystems was accurately predicted with just the incident light wavelengths typical of their environment as inputs (Arp et al., 2020).

Knowing the origins and motives behind these organisms operating the way they do is important in biological process development, particularly when engineering an organism. Selecting an organism that functions on either oxygenic or anoxygenic photosynthesis depends upon the application an engineer is designing for. For example, if a product or process is sensitive to oxygen, such as enzymes used in the production of butanol (Lan and Liao, 2011), perhaps the process would be better suited to the anoxygenic PNSB. Or, if a process is being designed around the consumption of CO_2_ and it is not an ideal situation to provide a separate electron donor [which is required for PNSB to reduce CO_2_ (Larimer et al., 2004)], then perhaps the process would be better suited to cyanobacteria. In any case, photosynthetic organisms have evolved to sustain their own growth and metabolic processes necessary for survival not to maximize chemical production. They would, thus, need to be engineered to maximize their capacity for industrial process scales starting with solar energy capture.

## Engineering Photosynthesis

In photosynthetic organisms, it is estimated that as much as 75% of incident photon flux is in excess (Lüttge, 2011). As a result, they have evolved methods like safety valves to regulate, reroute, and dissipate the excessive electrons excited within their reaction centers (Scholes et al., 2011). If these electrons could be redirected toward an alternative pathway or if the bottleneck to their usage could be removed, there could be an uptick in the potential for commercial viability.

One of the most notorious bottlenecks in photosynthesis is the first enzyme in the carbon fixation cycle: ribulose-1,5-bisphosphate carboxylase-oxygenase, also known as RuBisCO. RuBisCo is a large enzyme that can only process about three molecules of CO_2_ every second, which is a fraction of the substrate a typical enzyme can process (Alberts et al., 2014). Additionally, oxygen competes for its active site, which inactivates it for carbon fixation. Consequently, the energy of many excited electrons must be dissipated because they cannot be used fast enough. Many studies have, thus, been conducted to improve the efficiency of photosynthesis by improving the efficiency of RuBisCO using site-directed mutagenesis or directed evolution (Cai et al., 2014; Cotton et al., 2015). Efforts include increasing the selectivity of CO_2_ over oxygen as well as increasing the speed with which RuBisCO catalyzes its reactions (Cai et al., 2014; Cotton et al., 2015; Cummins et al., 2018). A recent analysis suggested that there is a trade-off between this specificity and turnover rate caused by selection pressures of the enzyme’s activity and stability (Cummins et al., 2018). Further understanding of this competition is needed in order to improve the constraints of RuBisCO. If carbon fixation is upstream of the target product production pathway, this step will be rate-limiting. Alternatively, incorporating an additional carbon-fixation enzyme can also reduce this bottleneck. In a recent study, phosphoenolpyruvate carboxylase, a carbon-fixation enzyme common in C4 and CAM plants, was inserted into the cyanobacterium *Synechocystis* PCC 6803 and resulted in an increase of ethylene production (Durall et al., 2020).

**FIGURE 2:**
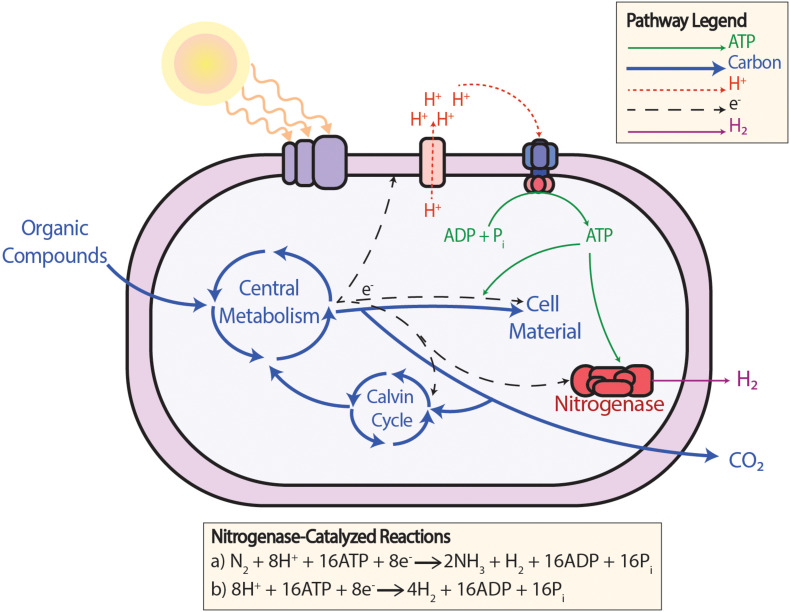
The production of H_2_ gas from the nitrogenase enzyme in PNSB under photoheterotrophic conditions. In wild-type PNSB, (a) the nitrogenase enzyme is expressed for N_2_ fixation when a more reduced nitrogen compound is not available. In mutant PNSB with the nitrogenase enzyme obligately expressed and more reduced nitrogen compounds are provided, (b) N_2_ does not need to be fixed and additional H_2_ can be produced using the same amount of ATP.

Bypassing carbon fixation by providing electrons with an alternative pipeline directly from the ETC is also a tactic being investigated. An example demonstrating this is the insertion of the mammalian cytochrome P450 CYP1A1 into cyanobacterium *Synechococcus* PCC 7002 as an artificial electron sink for excess electrons (Berepiki et al., 2016). In photoheterotrophic PNSB, such as *Rhodopseudomonas palustris* (*R. palustris*), hydrogen production is a natural alternative pathway to carbon fixation used as an electron sink (Figure 2). When the nitrogenase enzyme is obligately expressed, flux from the ETC preferentially flows toward hydrogen production over carbon fixation (McKinlay and Harwood, 2010, 2011). And, under certain circumstances, having an alternative pathway to carbon fixation is even a necessity for redox balancing. It was shown that mutant strains where RuBisCO was deleted could not grow at all due to a redox imbalance unless there was the alternative pathway toward hydrogen production the organism could use as an electron sink (Gordon and McKinlay, 2014). This shows that excess electrons can travel down this alternative pathway to hydrogen production.

The ETC itself has also been demonstrated to be a potential bottleneck to increased electron flux capacity. Investigating the difference between the cyanobacteria *Synechococcus* 7942 and the faster growing *Synechococcus* 2973, researchers concluded that there is a bottleneck following PSII: there are insufficient electron carriers to continue down the ETC in the slower growing strain— the faster strain had a higher expression of PSI, cytochrome b_6_f, and plastocyanin on a per cell basis (Ungerer et al., 2018). Similarly, in PNSB, using a computational genome scale metabolic model, it was shown that the quinol oxidation rate was a determining factor for energy production from the ETC (Alsiyabi et al., 2019). Thus, conceivably, the overexpression of complexes downstream of PSI would reduce the bottleneck in PNSB.

Another method being used to engineer organisms for industrial applications is adjusting the photochemistry of photosynthesis (i.e., engineering the complexes and antennae of light absorption). As mentioned previously, most of the photon flux incident on the light harvesting complexes are in excess. However, only a small portion of those photons are within the range of wavelengths absorbable by the antenna. In cyanobacteria, PSII absorbs wavelengths around 680 nm and PSI absorbs around 700 nm, which means they often compete for photons of similar wavelength (Blankenship et al., 2011). Thus, it is suggested that if they were absorbing from different regions of the electromagnetic spectrum with a wider electrical potential difference, efficiency would increase, which has been observed in photovoltaic cells (Hanna and Nozik, 2006; Blankenship et al., 2011). By introducing antennae from other organisms (Hitchcock et al., 2016), one can expand the range of different light wavelengths that can be used in oxygenic photosynthesis into the lower energy infrared region (Blankenship et al., 2011; Chen and Blankenship, 2011). Similarly, in anoxygenic organisms where there is only one photosystem, this could be achieved by modifying the antenna proteins surrounding the reaction center (Swainsbury et al., 2017). Increasing energy capture in photosystems could increase the potential for desirable chemical production in these organisms. The development of tools for the modification of photosystems could reduce rigidity in the wavelength of light necessary for function of these organisms (Swainsbury et al., 2017), such as in the context of artificial growth systems like a photobioreactor or perhaps in a mixed community of photosynthetic organisms that could be modified to absorb the same wavelength of light for simplicity of lighting design. With these tools, organisms could be decoupled from their environmental niche and adjusted with process design in mind.

Increasing the electron flux down the ETC increases the capacity for solar harvesting in photosynthetic bacteria and maximizes the generation of reducing equivalents— essentially optimizing the upstream process of a photosynthetic organism. Ultimately, this would be for naught if the downstream process is not also optimized. Whether generating a new pathway for product formation or increasing the formation of natural products, metabolic bottlenecks in the transfer of electrons in the upstream process to ultimate product formation need to be resolved.

## Engineering Electron Pathways for Product Formation

**FIGURE 3:**
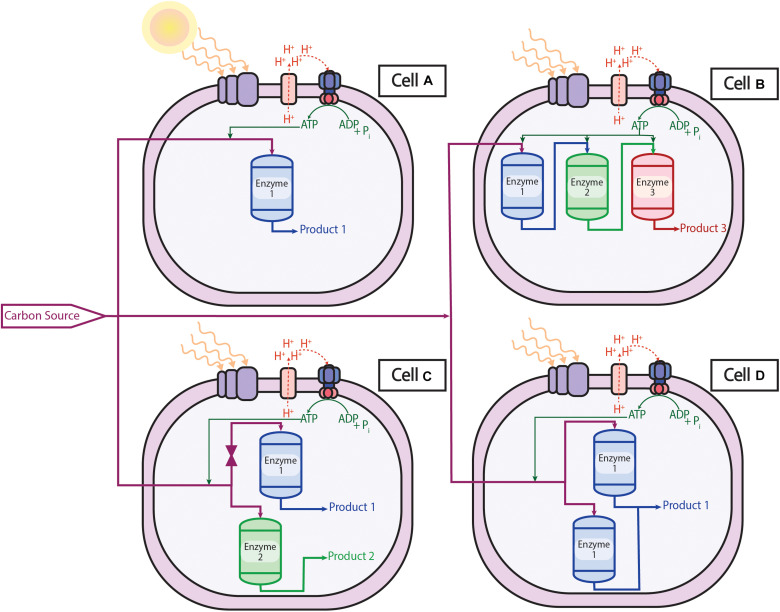
Solar-powered microbial cell factories: Cell A is the wild type organism that takes the carbon source, energy, and electrons and converts them to Product 1 via Enzyme 1. Cell B has had two heterologous enzymes (Enzyme 2 and Enzyme 3) inserted into the cell that produce Product 3 via the original Product 1. Cell C has silenced Enzyme 1 such that no or minimal Product 1 is produced in favor of Product 2 via the inserted non-native Enzyme 2. Cell D has overexpressed Enzyme 1 such that native Product 1 is overproduced.

Conventionally, genetic modifications used a combination of random mutations and phenotypic screening methods (Nielsen, 2001) but numerous sophisticated tools and procedures have since been developed and the field has expanded. Using synthetic biology tools like regulating transcription or translation and modifying genetic material through the insertion or deletion of genes (Cheng and Lu, 2012), one can repurpose microorganisms to produce or overproduce native and non-native chemicals in a completely renewable process. An engineer may wish to think of a cell as a chemical plant as illustrated in Figure 3. Parallels can be drawn between an operator opening and closing valves in the pipelines between reactors in a chemical plant to genetic modifications in a cell. The carbon sources and electrons of metabolism can be imagined as being transferred in a pipeline to a reactor to produce a specific product. In a cell, this reactor is an enzyme, whose expression can be controlled genetically.

The model organisms of metabolic engineering are the prokaryotic bacteria *Escherichia coli* (*E. coli*) and the eukaryotic yeast *Saccharomyces cerevisiae* (*S. cerevisiae*). They became model organisms mainly due to the speed at which they grow and the ease with which they can be handled but also because use begets more use. The more they were used for this application as the field was developing in the 1970s and 1980s, the more was known about them and the further technology was developed around them (Woolston et al., 2013). Other bacteria have also been used on a smaller scale for metabolic engineering for reasons such as their ability to grow at higher temperatures for faster reaction rates (e.g., thermophiles) or to use alternative feedstocks (e.g., CO_2_) (Woolston et al., 2013). However, in other organisms, synthetic biology tools have been reported to behave differently than they do in *E. coli* (Huang et al., 2010). Thus, since tool development in unconventional organisms has not been as rapid, the ability to use them in biological process development has also not been as rapid.

The engineering of photosynthetic organisms to produce heterologous products involves redirecting the electrons and energy generated from photosynthesis away from native pathways and toward more industrially advantageous products. Among photosynthetic microbes, cyanobacteria are the model organisms for metabolic engineering due to their genetic tractability and ability to produce valuable products using only CO_2_ and light (Jensen and Leister, 2014; Knoot et al., 2018; Xia et al., 2019; Hitchcock et al., 2020; Luan et al., 2020). As summarized in Table 1, the most common cyanobacterial strains used in metabolic engineering are *Synechococcus* 7942, *Synechococcus* 7002, and *Synechocystis* 6803, which have been used as chemical production chassis for such chemicals as biofuels, commodity chemicals, and flavoring compounds. One of the first successful heterologous pathways demonstrated in cyanobacteria was that of the biofuel ethanol (Deng and Coleman, 1999). A titer of 0.23 g/l was achieved by diverting the electron pathway from pyruvate for heterologous expression of the enzymes pyruvate decarboxylase and alcohol dehydrogenase from the organism *Zymomonas mobilis* (Deng and Coleman, 1999). As shown in Table 1, the titers of ethanol have since been improved 20-fold by a combination of gene overexpression, replacing native genes with better performing mutants, and improving cofactor availability along with some media optimization. Alternatively, pyruvate can be diverted to the production of different chemicals, such as the plastic precursor 2,3-butanediol by inserting the galactose-proton symporter and the enzymes G6P dehydrogenase and 6PG dehydrogenase (Kanno et al., 2017). However, electrons can be diverted from any point in the metabolism of an organism. Diverting electrons from further up the glycolytic pathway than pyruvate, such as from glucose, products such as sucrose can be produced (Ducat et al., 2012) or further down the pathway, such as from acetyl-CoA, fatty acids can be synthesized (Liu et al., 2011). While there have been advances in the development of tools for cyanobacteria (Wang et al., 2017), production of some of these chemicals are several orders of magnitude lower than they have been demonstrated to be in *E. coli*, due to relatively fewer synthetic biology tools that are effective in cyanobacteria, scarcity of plasmid libraries for expression of genes in cyanobacteria, and cyanobacterial polyploidy (Hitchcock et al., 2020; Luan et al., 2020).

**TABLE 1:** Cyanobacteria as cell factories: the production of heterologous products via genetic modification and the production titers thereof.

Organism	Product	Method	Titer	References
*Synechococcus 7942*	2,3-Butanediol	Insertion of *galP, zwf, gnd Overexpression of prk, rbcLXS* Deletion of *cp12*	12.6 g/L	Kanno et al., 2017
	Butanol	Insertion of *nphT7*, *pduP*, *phaB*, *phaJ*, *ter*, *yqhD*	0.4 g/L	Lan et al., 2013
	Ethanol	Insertion and overexpression of *pdc-adh* Deletion *glgC* Supplementation of cofactors (Mg^+2^, Zn^+2^, thiamine pyrophosphate, and NADP^+^)	3.9 g/L	Velmurugan and Incharoensakdi, 2020
	Glycerol	Insertion of *gpd1*, *hor2*	1.2 g/L	Hirokawa et al., 2016
	Isobutanol	Insertion of *alsS*, *ilvCD*, *kivD*, *yqhD* Deletion of g*lgC*	0.6 g/L	Li et al., 2014
	Isobutyraldehyde	Overexpression of RuBisCo Insertion of *alsS*, *kivD*, *ilvCD, rbcLS*	1.1 g/L	Atsumi et al., 2009
	Isoprene	Overexpression of *dxs* and *ispG* Fused proteins *IspS*-*IDI*	1.3 g/L	Gao et al., 2016
	Isopropanol	Insertion of *thlA*, *atoAD’*, *adc*, *adhE* Overexpressing *pdhABCD*	0.3 g/L	Hirokawa et al., 2020
	1,3-Propanediol	Insertion of *dhaB1*, *dhaB2*, *dhaB3*, *gdrA*, *gdrB, yqhD*	1.2 g/L	Hirokawa et al., 2017
	Squalene	Overexpression of *dxs*, *idi* Expression of *ispA* and *CpcB1*-*SF*-*SQS* fusion protein	12.0 mg/L	Choi et al., 2017
	Sucrose	Expression of *cscB* Deletion of *invA*, *glgC*	2.6 g/L	Ducat et al., 2012; Qiao et al., 2018
*Synechococcus 7002*	2,3-Butanediol	Insertion of *alsD*, *adh*	1.6 g/L	Nozzi et al., 2017
	Fatty acid	Insertion and overexpression *rbcLS*	0.1 g/L	Ruffing, 2014
	L-Lysine	Insertion of *ybjE, lysC*	0.4 g/L	Korosh et al., 2017
	Mannitol	Insertion of *mlp*, *mtlD*	1.1 g/L	Jacobsen and Frigaard, 2014
*Synechocystis 6803*	Acetone	Expression of *ctfAB*, *adc* Deletion of *phaCE*, *pta*	36.0 mg/L	Zhou et al., 2012
	Alkene	Overexpression of *sll0208* and *sll0209*	26.0 mg/L	Wang et al., 2013
	Ethanol	Insertion of *pdc*, *slr1192* Silencing of *slr9394*	5.5 g/L	Gao et al., 2012
	Fatty acid	Insertion of *tesA* Deletion of *slr1609*	0.2 g/L	Liu et al., 2011
	3-hydroxybutyrate	Insertion of *phaA*, *phaB1*, and *tesB* Deletion of *phaEC*	1.8 g/L	Wang et al., 2018
	3-Hydroxypropionic acid	Insertion of *mcr*, *pntAB*, *accBCAD, birA*	0.8 g/L	Wang et al., 2016
	Isobutanol	Insertion of *kivd, yqhD*	0.3 g/L	Miao et al., 2017
	Lactic Acid	Insertion of *ldh, pyk* Deletion of *ppc*	0.8 g/L	Angermayr et al., 2014
	Limonene	Overexpression of *rpi* and *rpe* Insertion of *gpps*	6.7 mg/L	Lin et al., 2017
*Synechococcus 2973*	Sucrose	Expression of *cscB* Overexpression of *sps* and *spp*	8 g/L	Lin et al., 2020

Facing similar difficulties, engineering the anoxygenic organism PNSB is an even more recent endeavor. However, interest in PNSB for its intrinsic ability to produce a commercially important product precedes efforts to engineer the organism itself. PNSB are naturally able to produce hydrogen gas as a by-product of nitrogen fixation and as an alternative pathway for an electron sink when there is an excess of electrons from photosynthesis (Gordon and McKinlay, 2014). This cellular process is shown in Figure 2. The initial efforts at metabolic engineering PNSB were mainly directed toward increasing hydrogen production through the deletion of competing pathways such as carbon dioxide assimilation (Joshi and Tabita, 1996), or through the obligate expression of nitrogenases such that hydrogen is produced even in the absence of nitrogen gas (Rey et al., 2007). Another product that both PNSB and cyanobacteria produce naturally is poly(3-hydroxybutyrate) or PHB (Ansari and Fatma, 2016; Ranaivoarisoa et al., 2019). PHB is a lipid used for energy storage in many prokaryotic bacteria but, as a marketable product, PHB is a polymer precursor for biodegradable plastics (Ranaivoarisoa et al., 2019). Engineering of these microbes resulted in titers of PHB as high as 1.9 g/l in PNSB (Kobayashi and Kondo, 2019) and 1.8 g/l in cyanobacteria (Wang et al., 2018).

More recently, interest in PNSB has shifted to other marketable products that are heterologous such as terpenoids, non-native carotenoids, and biofuels (Table 2). The sesquiterpenoids valencene (citrus flavor) and patchoulol (patchouli scent) are being produced in *Rhodobacter sphaeroides* on an industrial scale (Schempp et al., 2018). Carotenoids are commonly used as pigments, but they are also antioxidants that are used in health supplements (Fardoux et al., 2008; Wang et al., 2019). Since photosynthetic organisms naturally produce some carotenoids, much of the pathway is naturally present, from which carbon can be easily diverted to produce non-natural products of commercial importance such as lycopene and β-carotene (Giraud et al., 2018). Cyanobacteria are also used to produce carotenoids but since they are oxygen sensitive (Krinsky and Deneke, 1982), using anoxygenic organisms may be beneficial. Research in the production of biofuels other than hydrogen is a new area for PNSB with butanol being one of the most recent producing titers of 0.1 g/L from a butyrate feedstock (Doud et al., 2017).

**TABLE 2:** Purple non-sulfur bacteria as cell factories: the production of heterologous products and overexpression of native products via genetic modification and the production titers thereof.

Organism	Product	Method	Titer	References
*Rhodobacter Sphaeroides Rs265*	Amorphadiene	Expression of *ads* and MVA pathway (*mvaA*, *idi*, *hcs*, *mvk*, *pmk*, *mvd*) (Huembelin et al., 2014)	46.1 mg/L	Orsi et al., 2020a
	Coenzyme Q10	Overexpression of *crtE* and *ppsR* Deletion of *crtB*, *crtC*, *crtD* and *crtI*	73.2 mg/L	Zhu et al., 2017
	Lycopene	Insertion of *crtI_4_ and dxs* Deletion of *crtI*_3_, *crtC*, and *zwf*	66.0 mg/L	Su et al., 2018
	Sesquiterpene	Insertion of MVA pathway (*mvaA*, *idi*, *hcs*, *mvk*, *pmk*, *mvd*) (Huembelin et al., 2014) Deletion of *dxr*	7.0 mg/L	Orsi et al., 2020b
	Valencene	Insertion of *CnVS* and MVA pathway (*mvaA*, *idi*, *hcs*, *mvk*, *pmk*, *mvd*) (Huembelin et al., 2014)	0.4 g/L	Beekwilder et al., 2014
*Rhodobacter sphaeroides HY01*	Hydrogen	Overexpression of *atp* (F1 operon)	8.5 L_*H2*_/L	Zhang et al., 2016a
*Rhodobacter sphaeroides HJ*	Poly(3-hydroxybutyrate)	Insertion of *phaA3*, *phaB2*, and *phaC1* Deletion of *phaZ*	1.9 g/L	Kobayashi and Kondo, 2019
*Rhodobacter capsulatus SB1003*	Botryococcene	Insertion of *dxs*, *idi*, *fps*	0.1 g/L	Khan et al., 2015
	Hydrogen	Deletion of *mmsa*	4.7 L_*H2*_/L	Zhang et al., 2016b
	Patchoulol	Insertion of *P*, *ispA*, *dxs* and *idi*, and MVA pathway (*mvaA*, *idi*, *hcs*, *mvk*, *pmk*, *mvd*) (Huembelin et al., 2014)	24 mg/L	Troost et al., 2019
	Valencene	Insertion of *CnVS*, *ispA*, and MVA pathway (*mvaA*, *idi*, *hcs*, *mvk*, *pmk*, *mvd*) (Huembelin et al., 2014)	18 mg/L	Troost et al., 2019
*Rhodopseudomonas palustris TIE-1*	Squalene	Expression of *dxs* and a fused *crtE*-*hpnD* Deletion of *shc*	15.8 mg/g DCW	Xu et al., 2016
*Rhodopseudomonas palustris CGMCC 1.2180*	Methane	Mutation of *nifD* at *V75A* and *H201Q*	144 nmol/mg total protein	Ma et al., 2020
*Rhodopseudomonas palustris CGA009*	Butanol	Insertion of *adhE2*	0.1 g/L	Doud et al., 2017
	Hydrogen	Expression of *nifA* Deletion of *draT2*	1.3 L_*H2*_/L	Wu et al., 2016
*Rhodopseudomonas palustris CEA1001*	Canthaxanthin	Insertion of *crtY*, and *crtW* Deletion of *crtC* and *crtD*	0.8 mg/L	Giraud et al., 2018
	β-carotene	Insertion of *crtY*, and *crtW* Deletion of *crtC* and *crtD*	4.7 mg/L	Giraud et al., 2018
	Lycopene	Deletion of *crtC* and *crtD*	7.2 mg/L	Giraud et al., 2018
*Rhodospirillum rubrum S1*	Lycopene	Deletion of *crtC* and *crtD*	15 mg/L	Wang et al., 2012

The ability to produce marketable products on a small scale is an important step in the development of an industrial process but this process must be scalable. A common issue with failed process scale-ups is the development of a microbial strain without consideration for how suitable it would be for industrial production (Crater and Lievense, 2018). While there are several tactics for scale-up in conventional microbial systems based on oxygen mass transfer or volumetric power consumption (Marques et al., 2010), photosynthetic bacteria introduce additional bioprocess considerations.

## Engineering Light Delivery Systems

Engineering the internal chemistry of an organism to produce heterologous compounds or to increase the production of natural compounds is certainly essential in the development of economically viable bioprocesses. However, the external environment, in the form of reactor design and process optimization, also has a significant impact on product formation. Factors including light intensity and penetration as well as cell immobilization affect the metabolism of cells and, therefore, product formation.

One of the most important considerations in terms of reactor design for photosynthetic organisms is that of light delivery. Due to cell shading and light scattering, there is attenuation of light from the source into the depths of a reactor, particularly when the process liquid is turbid. Hence, delivering light uniformly and at an effective intensity can be a challenge. This challenge is one that the algae industry has been facing for many years. The most common system for algal growth is the outdoor raceway pond and other such open systems as they are the most economical and simple to operate (Nwoba et al., 2019). However, in addition to the issue of light attenuation, open systems are more difficult to scale up. Moreover, it is difficult to implement systems for pH or temperature control and maintain sterility in such systems (McBride and Merrick, 2014). When developing a process around an engineered organism, these factors are important to account for, and an open system would likely not be a practical or, ultimately, economical option if the engineered organism is outcompeted by wild bacteria. Developing methods for improving light delivery in a closed system has been a focus in the field of algal technology and one which has extended to use for other photosynthetic organisms (Heimann, 2016; Nwoba et al., 2019).

One of the techniques proposed for uniform light distribution is to grow cells on the surface on which light is incident using waveguide technology (Jung et al., 2012; Doud and Angenent, 2014; Genin et al., 2015; Delamare-Deboutteville et al., 2019; Nwoba et al., 2019). As the name suggests, a waveguide guides light from a light source and down the span of its surface allowing light to penetrate to the depths of a dark reactor and can also provide a surface to which cells can adhere. In addition to providing cells with light more effectively, using cell immobilization can be quite advantageous from an operational standpoint. Since biomass is already separate from the process fluid, downstream separation costs can be reduced through this method. Moreover, the immobilized biomass can be transferred to fresh media in a modular manner. For example, in a study using engineered yeast immobilized in a manufactured polymer, it was shown that cells could be reused for over a year of production (Johnston et al., 2020). In PNSB, *Rhodobacter capsulatus* cells immobilized on agar produced hydrogen stably for over 70 days (Elkahlout et al., 2019). In cyanobacteria engineered to produced ethylene, cells immobilized in an artificial biofilm matrix produced ethylene for up to 38 days (Vajravel et al., 2020). The reuse of cells without a growth phase could reduce yield losses incurred during the growth of the organisms.

Most bacterial cells naturally aggregate in a biofilm by excreting an extracellular polymeric matrix to adhere to surfaces and to each other (Halan et al., 2012). In nature, biofilms are one of the most widely distributed modes of life (Flemming et al., 2016). There are many benefits to biofilm cultivation such as the facilitation of intercellular interaction, resource capture, protection from external stressors like pH or temperature variations, and resistance to toxins (Flemming et al., 2016) that could prove advantageous while designing a robust host system for bioprocesses. This type of cellular immobilization has even been suggested as a means of improved reactor productivity because a higher density of cells can be concentrated into a smaller space than with suspended cultures and also because chemical products can diffuse out reducing toxicity of high chemical concentrations for the cell (Karagöz et al., 2009; Winkelhausen et al., 2010). This would mean increased product from the same reactor size as a suspended culture and has been shown to be the case for hydrogen production from PNSB (Chen and Chang, 2006; Adessi and De Philippis, 2014; Tsygankov and Kosourov, 2014; Wu et al., 2016; Adessi et al., 2017). It has also been suggested that, when in a microbial mat aggregate, cyanobacterial cells can export excess electrons to other cells in order to prevent over-reduction of their own photosystems ensuring that even cells with access to only lower energy wavelengths have access to electrons (Babauta et al., 2014; Lea-Smith et al., 2016). This suggests that cellular immobilization on the surface of a waveguide light source could encourage using neighboring cells as electron sinks for excess electrons in the event of production saturation, which would perhaps result in reduced photodamage and loss of energy as heat.

In addition to providing energy, light can be used as an engineering technique. Considering that the full spectrum of light is not absorbed by a photosynthetic organism and that different photosystems have evolved to preferentially absorb certain wavelengths and light intensities (Arp et al., 2020), light of different wavelengths can be used to modulate gene expression. Using monochromatic LED lights emitting wavelengths at the absorption maxima of a specific chromophore, one could select for different components of a photosystem. In the cyanobacteria *Arthrospira maxima*, more chlorophyll *a* can be formed under red light while phycobiliprotein reaches a maximum in blue light (Park and Dinh, 2019). Comparably, in the PNSB *R. palustris*, carotenoids can be produced maximally under blue light (Kuo et al., 2012).

Similar to the effects of wavelength, light intensity also affects gene expression in a photosynthetic organism. PNSB grow additional light harvesting complexes (pigments and carotenoids) around their photosynthetic reaction centers in the presence of low light intensity (Fassioli et al., 2009; Scholes et al., 2011). These additional chromophores increase the cross-section of light absorbable by the antenna and, thus, the energy which can be transferred to the reaction center for electron excitation. For example, it has been shown that in low light intensity (4 μmol photons/m^2^/s), *R. palustris* upregulates expression of different light harvesting complexes than it does under high light intensity (30 μmol photons/m^2^/s) (Fixen et al., 2019). This is caused by a change in the redox potential of the electron transport chain: under conditions of low light intensity, the quinone pool is more oxidized, which causes a redox signal (Fixen et al., 2019). Redox signals caused by a change in light intensity have also been observed in other PNSB (Swem et al., 2006; Wu and Bauer, 2010). Similar results are observed in cyanobacteria: in high light intensities, a higher growth rate is observed, and, in low light intensities, there is increased expression of light harvesting complexes (Jahn et al., 2018). From a practical perspective, one could potentially use this in process development: if the goal of the process is to produce pigments, one could ensure the photobioreactor has a low light intensity to increase their concentration. Conversely, increasing light intensity could result in an abundance of electrons that could be used for downstream chemical production. In PNSB, this has been studied with its native hydrogen production pathway where an increase in hydrogen is indeed observed with increased light intensity until saturation is reached (Uyar et al., 2007; Adessi and De Philippis, 2014). However, while an increased light intensity would increase electron availability, it is also associated with increased photodamage (Mellis, 1999). Increasing the rate of photodamage would result in reduced photosynthetic efficiency while the cell works to repair itself, so this is a consideration to make in the process design.

While bioreactors are often considered merely vessels for cellular growth, the way they are designed and operated affect the internal processes of the cell through upregulation or downregulation of genes in response to their environment. The availability, intensity, and wavelength of light inside a photobioreactor affect the structure and quantity of solar harvesting complexes for photosynthesis, which impacts the energy and electrons available for downstream cellular processes. Likewise, cellular immobilization can make cells more resilient to sudden changes in temperature, pH, and nutrient availability by creating a microenvironment separate from the reactor bulk fluid and where there is a division of labor among the individual cells (Flemming and Wingender, 2010). All aspects in the design of a bioprocess affect each other and, while this interconnectedness increases complexity, it also implies countless opportunities for optimization and improvement as well as possibilities for product formation.

## Conclusion

To “begin with the end in mind” is an approach recommended when the overall goal is large-scale production (Crater and Lievense, 2018). More specifically, it is the idea that the scale-up of a process into industrial production will influence research from the beginning in the selection of the product and microbe and throughout the research process. In the case of solar-powered microbial cell factories, the selection of a photosynthetic organism depends largely on the chemical desired as a product and the feedstocks available as inputs. This necessitates an understanding of the internal cellular processes from photosynthesis to central metabolism and the effects of environmental factors on both.

A simple explanation of the differences between oxygenic and anoxygenic photosynthetic organisms is that oxygenic photosynthesis, such as that performed by cyanobacteria, evolves oxygen from the splitting of water molecules whereas anoxygenic photosynthesis, such as that performed by purple bacteria, does not evolve oxygen. Additional distinctions include the differing complexes of the ETC, whether the flow of electrons within the ETC is cyclic or not, and differences in feedstock preferences: some organisms more efficiently consume CO_2_ while others specialize in the uptake of organic carbons in wastewater. However, there is more complexity to it when considering the internal processes and bottlenecks in the ETC.

With much of the energy incident on photosynthetic organisms being dissipated as waste, there is considerable opportunity to increase the flux of electrons through the ETC and toward the production of chemicals. One bottleneck is RuBisCO, which is both slow and lacking in selectivity for the fixation of carbon. Another is the complexes in the ETC itself, where increasing expression of electron carriers can improve electron flux capacity. A further prospect to augment electron flux through the ETC is to modify the antennae of photosynthesis to increase the spectrum of absorbable energy. With more energy and electron flux available from the ETC, there is an enhanced ability to produce chemicals.

The production of chemicals using strategies such as the expression of heterologous genes, the deletion of native genes, and the overexpression of genes has been demonstrated in both oxygenic and anoxygenic photosynthetic organisms, but on significantly lower scales than with model organisms such as *E. coli* and yeast. Development and improvement of the library of tools available for use in unconventional organisms will be needed to advance this field, as will be the development of photobioreactors practical for this application on a large scale.

Considerations for light availability inside a photobioreactor is a challenge for effective distribution and for setpoint optimization both for wavelength and intensity. These affect the structure and availability of photosynthetic complexes and thus the efficiency of energy harvesting. The development of strategies for effective light distribution is an area requiring advancement. Another technology that requires further development is the coupling of light to solar energy whether through waveguides that guide solar light or through solar-powered lighting systems.

Both oxygenic and anoxygenic photosynthetic organisms have vast potential to change the course of consumerism to one that is environmentally conscious. However, diverting electrons and energy stored from photosynthesis toward chemical production pathways is no small feat. The production of chemicals from photosynthetic organisms has been demonstrated but, for the most part, currently lacks the titers and the equipment necessary for large-scale production. While microbial cell factories have been in development for more than 40 years and some have reached industrial quantities (Yim et al., 2011), the advancement of solar-powered microbial cell factories has been delayed largely by a lack of genetic tools and challenges associated with photobioreactors such as the delivery of light. Considering all these factors in a holistic approach will likely be necessary to reach industrial scales of production. The potential to use solar energy to power the production of chemicals from anthropogenic waste will be critical to address net zero emissions in most advanced economies.

## Author Contributions

SS compiled the literature and drafted the manuscript. RM and DGA revised and edited the manuscript. All authors contributed to the article and approved the submitted version.

## Conflict of Interest

The authors declare that the research was conducted in the absence of any commercial or financial relationships that could be construed as a potential conflict of interest.
